# Thoracic endovascular aneurysm repair to treat recurrent lower limb ischemia secondary to occlusion of axillofemoral bypass

**DOI:** 10.1016/j.ijscr.2020.02.053

**Published:** 2020-02-28

**Authors:** Mototsugu Tamaki, Hideki Kitamura, Yutaka Koyama, Koshi Sawada, Yasuhiko Kawaguchi, Takahiro Tokuda, Yasuhide Okawa, Kazuya Konakano

**Affiliations:** Department of Cardiovascular Surgery, Nagoya Heart Center, Nagoya, Japan

**Keywords:** TEVAR, Chronic aortic dissection, Malperfusion, Entry closure

## Abstract

**Introduction:**

A case of malperfusion in which the patient presented with aortic dissection is presented.

**Presentation of case:**

A 69-year-old man with an acute aortic dissection (Stanford type B) had lower limb ischemia. Axillary-femoral bypass was performed, and his lower limb ischemia improved. Eight months after the onset of acute aortic dissection, he again had lower limb ischemia. Contrast-enhanced computed tomography showed axillary-femoral bypass occlusion and true lumen collapse, compressed by the increased false lumen pressure in the aorta. Thoracic endovascular aortic repair (TEVAR) was performed for entry closure. His lower limb ischemia was improved by TEVAR.

**Discussion:**

One of the complications of type B aortic dissection is malperfusion. Endovascular therapy is a first step in treating the malperfusion of type B aortic dissection. It is important to seal the entry for the treatment of malperfusion.

**Conclusion:**

If there is an entry, it is important to seal it for the treatment of malperfusion.

## Highlights

• One of the complications of type B aortic dissection is organ ischemia.
• TEVAR was performed for entry closure.
• TEVAR improved malperfusion.

## 1 Introduction

The primary treatment of acute aortic dissection (Stanford type B) remains medical. However, patients with life-threatening complications of acute aortic dissection (such as rupture, malperfusion, or uncontrollable pain) require emergency treatment using open surgical aortic graft replacement, thoracic endovascular aortic repair (TEVAR), fenestration, or extra-anatomic surgical bypass [1]. In the case presented, TEVAR was performed for limb ischemia due to malperfusion caused by chronic aortic dissection with axillofemoral bypass occlusion.

This work has been reported in line with the SCARE criteria [2].

## 2 Case presentation

An obese, 69-year-old man with hypertension and diabetes mellitus suddenly complained of back pain. He was diagnosed with acute aortic dissection (Stanford type B) at another hospital by contrast-enhanced computed tomography (CECT) and was transferred to our hospital by ambulance.

**Fig. 1:**
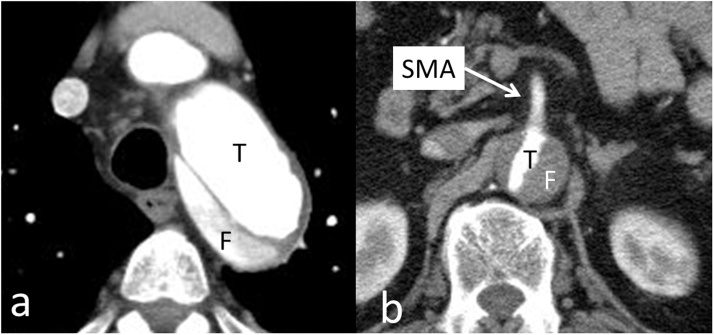
The true lumen is compressed by the false lumen. **a**. axial slice at the level of the distal arch, **b**. axial slice at the level of the superior mesenteric artery (SMA). **T**: true lumen, **F**: false lumen.

CECT showed a Stanford type B aortic dissection starting just distally to the left subclavian artery and extending to the renal artery. The pressurized false lumen encroached on the true lumen of the aorta, and the abdominal aorta was compressed by the false lumen (Fig. 1). Since he continued to have lower limb ischemia during medical therapy, right axillary-right femoral bypass was performed. His lower limb ischemia and general status improved, and he was discharged from our hospital.

**Fig. 2:**
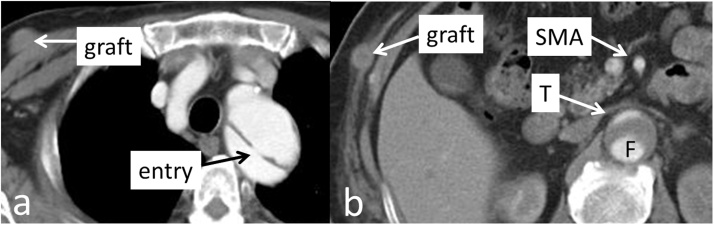
The true lumen is compressed by the false lumen. It is unchanged from before. However, the axillary-femoral bypass is occluded. **a**. axial slice at the level of the distal arch, **b**. axial slice at the level of the superior mesenteric artery (SMA). **T**: true lumen, **F**: false lumen.

Eight months after the onset of acute aortic dissection, he developed lower limb pain after walking 50 m. The dorsalis pedis artery was palpable at rest bilaterally, but it was not palpable after a short walk. CECT showed occlusion of the axillary-femoral bypass, but the true and false lumen findings were the same as eight months earlier (Fig. 2). Since the blood pressure was low at the time of CT, there was no change. However, when the blood pressure was high at the time of body movement, the true lumen collapsed due to compression by the increased false lumen pressure in the aorta.

**Fig. 3:**
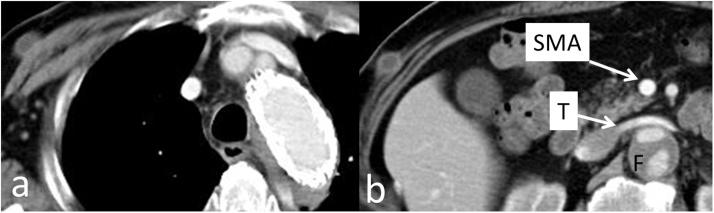
Blood flow through the true lumen is improved. **a**. axial slice at the level of the distal arch, **b**. axial slice at the level of the superior mesenteric artery (SMA). **T**: true lumen, **F**: false lumen.

TEVAR was performed for entry closure under general anesthesia. The optimal proximal landing zone for the TEVAR graft was 2 cm from the entry. Left internal carotid artery-left subclavian artery bypass with a Gelsoft graft (diameter 8 mm, Vaskutek Ltd, Inchinnan, UK) was performed first. Since the aortic diameter was different, a 28 mm × 100 mm Conformable TAG (W.L. Gore & Associates, Inc., Newark, DE, USA) was deployed to the descending aorta. Then, a 37 mm × 100 mm Conformable TAG was deployed just distal to the left internal carotid artery to seal the entry. Postoperatively, intermittent claudication disappeared, and he could walk more than 500 m. His lower limb ischemia was improved by TEVAR. Seven days after the operation, CT showed improved blood flow in the true lumen (Fig. 3).

He was discharged on the 11th postoperative day, and he is doing well one year after the operation.

## 3 Discussion

The treatment of uncomplicated type B aortic dissection remains medical [3]. However, type B aortic dissection sometimes has life-threatening complications. One of the complications of type B aortic dissection is malperfusion. About 4.7% of patients present with peripheral malperfusion. Lower limb ischemia requires immediate open surgical or endovascular management [1,3].

The patient had lower limb ischemia when he developed acute aortic dissection (Stanford type B) and underwent right axillary-right femoral bypass. However, he developed ischemic complications 8 months after the onset of acute aortic dissection. At the time of a rise in blood pressure, the flow through the true lumen decreased, but at the time of a drop in blood pressure, the flow through the true lumen improved. So, when the flow of the true lumen improved, the bypass flow was not needed. Therefore, we suspected that the bypass was occluded. This clinical course was unusual, because it took a long period of 8 months for the symptoms to appear after the occurrence of aortic dissection.

One of the causes of malperfusion is increased false lumen pressure. The true lumen collapsed because of compression caused by the increased false lumen pressure at the entry in the present case. Therefore, it was thought that entry closure was necessary. The treatment for malperfusion is surgical or endovascular. Endovascular therapy is a suitable first step in treating type B aortic dissection to reduce early morbidity, mortality, and hospitalization [4]. Wilkinson et al. reported that TEVAR (80%) and open descending aortic repair (82.8%) were similar in 5-year freedom from aortic re-intervention or rupture [5]. In the present case, axillofemoral bypass was initially performed as the preferred procedure because we did not have the infrastructure or expertise to perform TEVAR. Furthermore, the patient’s clinical condition did not permit transfer to another hospital for TEVAR. However, peripheral malperfusion returned. At that point, it was judged that endovascular treatment was appropriate for entry closure. Since the symptom of lower limb ischemia was not severe, he was able to wait for treatment. Additionally, we had gained technical expertise and developed the TEVAR programme over the previous six months, enabling us to perform TEVAR on this admission.

The purpose of TEVAR is to seal the entry and decrease inflow into the false lumen [4]. If the pressure of the false lumen decreases, the pressure of the true lumen rises, and malperfusion is improved [4,6].

In the present case, right axillary-right femoral bypass was performed first, but the patient’s limb ischemia recurred. TEVAR was then performed to seal the entry. His intermittent claudication disappeared after TEVAR, and he was doing well one year after the procedure. Had TEVAR been performed first, his limb ischemia might not have recurred. This clinical course suggests that malperfusion presenting with limb ischemia is caused by increased false lumen pressure. Thus, it is important to seal the entry for the treatment of malperfusion.

## 4 Conclusion

In patients with dissection, if there is an entry, malperfusion can occur in the chronic stage. It is thus important to seal the entry.

## Sources of funding

No funding was received.

## Ethical approval

Exception from ethical approval – case report only, consent from the patient provided at request.

## Consent

The patient provided written, informed consent to the publication of this case report.

## Author contributions

MT wrote the manuscript. HK and YO supervised writing the manuscript. All authors were part of the surgical team that treated this patient. All authors read and approved submission of the final manuscript.

## Registration of research studies

Not applicable.

## Guarantor

Mototsugu Tamaki.

Hideki Kitamura.

## Provenance and peer review

Not commissioned, externally peer-reviewed.

## Declaration of Competing Interest

The authors declare that there is no conflict of interest.
